# Obesity: Policy and Practice Recommendations for High-Risk Populations Influenced by the COVID-19 Pandemic

**DOI:** 10.1128/msystems.00089-22

**Published:** 2022-05-31

**Authors:** Kathryn N. Robinson, Deborah A. Saber

**Affiliations:** a School of Nursing, UMaine Graduate School, University of Maine, Orono, Maine, USA; b UMaine Institute of Medicine, University of Maine, Orono, Maine, USA; c Maine Cancer Genomics Initiative, The Jackson Laboratory, Bar Harbor, Maine, USA; d School of Nursing, University of Maine, Orono, Maine, USA; e Nursing Research and Evidence-Based Practice, Northern Light Eastern Maine Medical Center, Bangor, Maine, USA; University of Maine

**Keywords:** obesity, inflammation, policy, recommendations, novel virus, resilience, COVID-19, SARS-CoV-2, social determinants of health

## Abstract

Obesity has been linked to higher morbidity and mortality in those that contract novel viruses, such as H1N1 and SARS-CoV-2. Loss of life and the high cost of obesity highlight the need to focus on preventative measures. The state of Maine (United States) is profiled as a rural state with high rates of obesity and low health literacy that benefits from programs to improve health. However, more multidirectional efforts are needed. Four recommendations for building a healthier, more resilient patient population are discussed here: (i) state incentives and subsidies, (ii) employers to incentivize healthy living, (iii) federal incentives and initiatives, and (iv) personal responsibility for health and wellness.

## PERSPECTIVE

A driving force that promotes health and prevention of disease among high-risk, vulnerable populations is the continued development of ecological approaches at the individual and population levels. However, comorbid conditions or diseases, such as obesity, can predispose an individual to high-risk infectious diseases such as SARS-CoV-2, with increased severity in complications that make interventions more complex (1). This cascading effect is further influenced by changes in an individual’s social determinants of health that can lead to more significant health disparities. Sometimes these modifiable risk factors can result in harmful responses with exposure to infectious diseases, leading to poor health outcomes, including death (2).

Obesity has historically been classified as a disease, but with increased understanding it is now referred to as a condition or comorbid condition and underserved, rural populations are targeted as high risk. As the COVID-19 pandemic has highlighted, strategies to improve overall population health are necessary to promote a prepared and resilient society. This article will discuss obesity as a comorbid inflammatory condition and provide recommendations to promote strategies for wellness.

## OBESITY AS A COMORBID CONDITION

A common comorbid condition that has been linked to infectious diseases is obesity (3, 4). This condition creates a persistent pathological inflammatory process defined as a “response to infection, antigen challenge or tissue injury designed to eradicate microbes or irritants and to potentiate tissue repair” (p. 386) (5). The chronic proinflammatory state present in adipose tissue predisposes obese patients to poor health outcomes when another inflammatory condition (e.g., virus) is introduced. In patients with H1N1, Louie et al. (2) found a link between obesity and poor outcomes and higher mortality for those with a body mass index (BMI) above 45 kg/m^2^ (odds ratio [OR] 4.2; confidence interval [CI] = 1.9 to 9.4). Most recently, these findings were supported during the COVID-19 pandemic. Increased hospitalizations tripled in SARS-CoV-2-infected obese patients (6). Likewise, this increase was independently attributed to physical inactivity and smoking (7). Among 900,000 adult COVID-19 hospitalizations in the United States, over 30% patients had obesity as a comorbid condition (6). Moreover, the severity of COVID-19 illness has been strongly linked to the severity of disease, the cost of care, and the number of lives lost (8–11). In a health care cost model, the authors found that 20.3% of patients with a BMI of >40 kg/m^2^ required intensive care treatment, including invasive mechanical ventilation compared to 6.6% of those with BMI of <25 kg/m^2^ (12). The economic cost of treating obese patients with COVID-19 nearly doubled for those with a BMI of >40 kg/m^2^ compared to those with a BMI of <25 kg/m^2^ (12).

Although genetic composition can contribute to BMI, obesity is primarily considered a worldwide preventable—or modifiable—epidemic (13, 14) with widespread, long-lasting implications. The problem is escalating. The World Health Organization reported that the prevalence of overweight or obese children and adolescents aged 5 to 19 years increased >4-fold from 4 to 18% globally from 1975 to 2016 (15). Of U.S. adults, 42.5% are classified as obese (BMI ≥ 30 kg/m^2^) and 9% as severely obese (BMI > 40 kg/m^2^) (16). This alarming epidemic predisposes children and adults to additional comorbidities that include diabetes mellitus and hypertension, adding a higher likelihood of poor outcomes and mortality with the introduction of novel viruses (1). To address and work toward reducing this upward trajectory of obesity, several national agencies have developed recommendations to review the role of social inequities.

The Office of Disease Prevention and Health Promotion aims to achieve health equity by addressing determinants of health, defined as “the range of personal, social, economic, and environmental factors that influence health status,” having a profound impact on health outcomes (17, 18). Social determinants of health (SDOH) reflect the environment’s social factors and physical conditions, which are influenced by the geographic location, social services, and socioeconomic status of vulnerable individuals residing in communities. Moreover, the current health care landscape and challenges that impede health are impacted by SDOH.

One far-reaching and sometimes crippling SDOH is health literacy, described as having the adequate ability to read and comprehend essential health-related materials. Good literacy is necessary to achieve healthy-lifestyle management skills that are aimed at reducing rates and complications of chronic illnesses such as obesity (18–20). According to the U.S. Department of Health and Human Services in 2021, nearly 9 of 10 adults struggle with health literacy (21), and only 12% of the U.S. population are currently considered to have high health literacy (22). Several factors influence an individual’s level of health literacy, such as socioeconomic, cultural, and psychosocial behaviors. Moreover, those with more significant health disparities (e.g., those living in a rural state) with low income and education status have been associated with lower health literacy levels (23).

## CURRENT STATE OF MAINE PEOPLE AND COMMUNITY NEEDS

One rural U.S. state with high health literacy challenges is Maine. Maine faces a health crisis from a high prevalence of chronic diseases, including obesity, complicated by many socioeconomic and sociocultural factors, including an aging population with high poverty rates. Chronic diseases are now the leading cause of death in the state of Maine (24) and rank ninth in the country overall (25). Obesity rates have consistently been over 64% for the past decade, and close to 60% are classified as either overweight or obese (24). From 2011 to 2016, the obesity rate (>30 BMI) in Maine increased from 27.8 to 29.9% (26). In addition, 13.7% of the youth population (ages 10 to 17) and 14.6% of children (ages 2 to 4) are considered obese (27). Health literacy deficits intensify the decreasing overall health of a state’s population (23). In Maine, 10.9% of the population is living at or below the poverty line (28, 29), of which 2.6% have less than a 9th-grade education (30). Given these challenges, the Maine Bureau of Health continues to argue that more proactive policies are needed at the local, state, and national levels to alleviate these top health issues (24).

Maine is the oldest, most rural state (31, 32) and is facing a crisis in chronic diseases at an epidemic level, leading the CDC-SDOH to develop frameworks that place additional focus on food and nutrition security (33, 34). SDOH frameworks have been used for public surveillance (33, 35, 36) and have focused on identifying individuals considered high risk, especially among disparate populations. Therefore, any recommended initiatives, new or revised policies should be tailored to meet the needs of the Maine people.

## RECOMMENDATIONS FOR PREPAREDNESS

Proactive campaign approaches (e.g., antismoking) have been used to improve health outcomes with various levels of success. Like previous efforts to improve health and reduce the health care system burden, successful strategies require a multitiered approach from local and state agencies, the food industry, and employers. Next, four recommendations incorporating government, community, and individual efforts provide a framework to promote well-being and resilience when faced with novel viruses.

## #1: AVAILABLE STATE INCENTIVES AND SUBSIDIES BUT MORE IS NEEDED

During the COVID-19 pandemic, the Maine people understood that collaborating with health care systems and state agencies for medical and community guidance led to being one of the top states with the lowest infection rates until late 2021 (37). State-led services covered the costs of COVID-19 testing and vaccinations, including those without health insurance (38). Other avenues for aid to high-risk and vulnerable populations included free internet service and financial assistance through the Maine Low Income Home Energy Assistance Program. However, only a few state incentives and subsidies exist focusing on the epidemic of obesity. One proactive approach is the Healthy Eating Active Living (HEAL) initiative, which prioritizes the goal to decrease the prevalence of obesity and the impact of related chronic conditions (26). The Maine Health Let’s Go Program is a multisetting obesity prevention initiative in Maine and New Hampshire targeting school nutrition and education programs and health care practices to encourage the adoption of healthy eating and lifestyle habits (39). Some states are helping feed people while working toward reducing food to landfills through laws that mandate the distributors to donate food rather than dispose of it as waste (40). Virtual opportunities are needed to provide education to rural areas and financial grants for those living in poverty and struggling with food security.

## #2: STRATEGIES FOR EMPLOYERS TO INCENTIVIZE HEALTHY LIVING

Employers can be powerful advocates for healthy living. Many businesses currently offer wellness programs and incentives for healthy living that promote mental health and nonsmoking. These programs benefit employees through financial rewards and cost savings from a healthy employee pool (41). Additional incentive programs could promote healthy weight maintenance, weight loss, and wellness tiers that focus on healthy eating and weight management (e.g., reimbursement for regularly attended exercise programs). The 2010 Affordable Care Act encourages organizations to adopt wellness programs by letting them offer participation incentives up to 30% of the total cost of health insurance coverage (42). For eligibility, companies can require educational sessions to improve health or active participation in weight-loss programs (43). In addition, healthy snacks could replace high sugar, high-fat items in employee vending machines, cafeteria foods could cater toward healthy eating, and “optimal health” clinics could be charged with high visibility campaigns to promote healthy weights through educational programs are tied to insurance incentives or bonuses.

## #3: TAKE ADVANTAGE OF FEDERAL INCENTIVES AND INITIATIVES

During the COVID-19 pandemic, U.S. consumers significantly changed their food-spending patterns as supply chains were disrupted, political instability increased, unemployment rates skyrocketed, and nonessential businesses closed (44). Federal subsidies exist that provide financial relief and food security in three ways. (i) Maine farms, which encompass 1.3 million acres (45), could receive federal aid through the Coronavirus Food Assistance Program, which provided $7.8 million in Maine relief in 2020 (46). However, Federal subsidies were distributed to the largest producers of corn, soybeans, wheat, cotton, and rice (45), and Maine farms produce mainly dairy products, livestock, and vegetables (47). Local and state agencies could feature organically rich food to promote wellness and disease prevention. (ii) During the pandemic, the U.S. Department of Agriculture covered certification and education expenses to farms producing certified organic or transitioning to organic farms (48). Maine communities can provide fresh, organic foods to food markets and grocery stores and encourage community gardens. Also, a licensing law agreement with the federal program, the Child and Adult Care Food Program (CACFP) provides nutritious meals and snacks to children and adults in their care through federal funding (49). However, only 45% (*n* = 1617) of licensed childcare programs in Maine utilized CACFP in 2021. These types of programs can be promoted. (iii) Finally, there are 86 census tracts designated as food deserts in Maine (49), where affordable or good-quality fresh food is difficult to buy. Maine health care agencies continue to promote a healthier lifestyle through “Healthy People 2020,” a federal government prevention agenda for “building a healthier nation” by increasing public awareness and education surrounding physical activity and tracking the proportion of children, adolescents, and adults with obesity (50). Prioritizing SDOH among high-risk populations is essential in developing the best approaches for initiatives focusing on physical activity and nutrition.

## #4: PERSONAL RESPONSIBILITY FOR HEALTH AND WELLNESS

Consumers have a broad understanding of what encompasses a wellness approach. Promoting wellness behaviors for preventable, chronic medical conditions can provide health and well-being outcomes from a health care perspective. From a consumer’s perspective, the meaning of health and wellness is evolving as more scientific facts emerge and are influenced by a shifting political and socioeconomic climate. Like the 1980s antismoking campaigns, citizens need to be educated on the link between obesity and illness. A reshaped health and wellness landscape will continue to impact consumers’ fundamental wellness views. The goal is to increase health literacy in individuals and empower them to make well-informed health-related decisions, which will lead to a healthier, happier lifestyle. “Digital” health literacy could help people toward making informed health care decisions. Increasing access to electronic sources such as websites and gaining access to personal health information will provide quicker involvement (51). When consumers have easily accessible and free healthy opportunities, they may be motivated to participate.

## CONCLUSION

Health-related initiatives have been discussed. The recommendations proposed can be used as a framework to decrease obesity and increase wellness. Novel infections will continue to emerge, and it is critically important to reduce obesity (and all inflammatory diseases) to improve the health of the country and the world. Our obesity crisis should be at the forefront of health care.
